# CCR2-overexpressing mesenchymal stem cells targeting damaged liver enhance recovery of acute liver failure

**DOI:** 10.1186/s13287-022-02729-y

**Published:** 2022-02-05

**Authors:** Ruixuan Xu, Beibei Ni, Li Wang, Jiarou Shan, Lijie Pan, Yizhan He, Guo Lv, Huizhu Lin, Wenjie Chen, Qi Zhang

**Affiliations:** 1 Biotherapy Centre, The Third Affiliated Hospital, Sun Yat-Sen University, 600# Tianhe Road, Guangzhou, 510630 China; 2 Cell-Gene Therapy Translational Medicine Research Centre, The Third Affiliated Hospital, Sun Yat-Sen University, Guangzhou, China; 3 Guangdong Provincial Key Laboratory of Liver Disease Research, The Third Affiliated Hospital, Sun Yat-Sen University, Guangzhou, China; 4 Department of Hepatic Surgery and Liver Transplantation Centre, The Third Affiliated Hospital, Sun Yat-Sen University, Guangzhou, China

**Keywords:** Acute liver failure, Mesenchymal stem cells, CCL2, CCR2, Homing

## Abstract

**Background:**

Mesenchymal stem cell (MSC) transplantation is emerging as a promising cell therapeutic strategy in acute liver failure (ALF) clinical research. The potency of MSCs to migrate and engraft into targeted lesions could largely determine their clinical efficacy, in which chemokine/receptor axes play a crucial role. Unfortunately, the downregulation of chemokine receptors expression after in vitro expansion results in a poor homing capacity of MSCs.

**Methods:**

By evaluating the chemokine expression profile in the liver of ALF patients and ALF mice, we found that CCL2 expression was highly upregulated in damaged livers, while the corresponding receptor, CCR2, was lacking in cultured MSCs. Thus, we genetically modified MSCs to overexpress CCR2 and investigated the targeted homing capacity and treatment efficacy of MSC^CCR2^ compared to those of the MSC^vector^ control.

**Results:**

In vivo and ex vivo near-infrared fluorescence imaging showed that MSC^CCR2^ rapidly migrated and localized to injured livers in remarkably greater numbers following systemic infusion, and these cells were retained in liver lesions for a longer time than MSC^vector^. Furthermore, MSC^CCR2^ exhibited significantly enhanced efficacy in the treatment of ALF in mice, which was indicated by a dramatically improved survival rate, the alleviation of liver injury with reduced inflammatory infiltration and hepatic apoptosis, and the promotion of liver regeneration.

**Conclusions:**

Altogether, these results indicate that CCR2 overexpression enhances the targeted migration of MSCs to damaged livers, improves their treatment effect, and may provide a novel strategy for improving the efficacy of cell therapy for ALF.

**Supplementary Information:**

The online version contains supplementary material available at 10.1186/s13287-022-02729-y.

## Introduction

Acute liver failure (ALF) is a life-threatening condition with a high mortality rate caused by a variety of factors, such as viral infection, drug-induced hepatotoxicity, and autoimmune or metabolic disease, whereas the available therapies are limited [1]. Currently, orthotopic liver transplantation (OLT) is widely approved as the most effective therapy for patients with ALF. However, the shortage of donor livers, immune rejection response, and complications caused by lifelong immunosuppressive treatment severely limit the clinical application of OLT. Thus, the investigation of alternative therapeutic strategies for ALF is highly desired.

Recently, mesenchymal stem cell (MSC) transplantation has emerged as a promising candidate for cell therapy in regenerative medicine. Due to their self-renewal, multipotent differentiation, and immunoregulatory characteristics and because they can be easily isolated and expanded in vitro [2–5], MSCs can be infused as a potential alternative treatment for ALF. Several previous studies have shown that MSC transplantation can promote recovery from liver injury [6, 7]. However, there are still certain obstacles that restrict the clinical efficacy of MSCs [8].

It has been shown that the treatment efficacy of MSCs depends largely on their capacity to engraft into lesions [9–12]. After systemic infusion, only a small portion of MSCs enter damaged tissues; the majority would be entrapped in the capillary beds of the lungs [13–15]. Inadequate engraftment of MSCs in damaged tissues may impair their clinical efficacy [16, 17]. Thus, it is imperative to enhance the targeted migration of MSCs toward lesions. MSC homing to the site of injury is mainly dependent on the interactions of chemokines from lesions with the corresponding chemokine receptors expressed by MSCs [18–20]. However, during continuous in vitro passaging, MSCs gradually lost their homing capacity as a result of the downregulation of chemokine receptors expression; in contrast, freshly isolated MSCs exhibit relatively good homing to damaged tissues [21, 22].

Therefore, modifying MSCs to overexpress chemokine receptors may be a strategy that improves the treatment effect of these cells. Several previous studies have genetically modified MSCs with specific chemokine receptors in an attempt to enhance their targeted homing capacity in different disease models. For instance, overexpression of CXCR5 markedly increased the homing of MSCs to the inflamed skin in mice and suppressed contact hypersensitivity [23]. Moreover, CXCR2-modified MSCs showed increased migration to inflamed mucosa and significantly accelerated ulcer healing in oral mucositis mice [24].

In this study, we found that CCL2 was intensely upregulated in the liver of ALF patients and ALF mice, while the corresponding receptor, CCR2, was lacking in MSCs. We hypothesized that genetically modifying MSCs to overexpress CCR2 would enhance the targeted migration of MSCs to the damaged liver, thereby improving their treatment effect. MSC migration and homing were dynamically monitored using in vivo near-infrared fluorescence imaging.

## Materials and methods

### Human liver specimens

Six damaged liver specimens were obtained from ALF patients receiving orthotopic liver transplantation in the Liver Transplantation Centre of the Third Affiliated Hospital of Sun Yat-sen University. Three normal liver specimens were obtained from donation after brain death (DBD) during liver transplantations. All clinical procedures were approved by the ethical committee of the Third Affiliated Hospital of Sun Yat-sen University. Informed consent was obtained from all individuals. Basic information of the ALF patients included in this study is summarized in Additional file 1: Table S1.

### Experimental animals and an ALF model

Male C57BL/6 mice, 8 weeks old, were purchased from the Model Animal Research Center of Nanjing University. The ALF model was induced by intraperitoneal injection of 300 mg/kg thioacetamide (TAA; Sigma-Aldrich, USA, 163678) as previously described [25]. Mice in the control group were intraperitoneally injected with an equal volume of saline. All animal experiments were carried out in accordance with the guidelines of the Animal Care and Use Committee of Sun Yat-sen University.

### Transplantation procedures

Mice were randomized into 4 groups: group 1, healthy control; group 2, ALF + PBS; group 3, ALF + MSC^vector^ (1.0 × 10^6^); and group 4, ALF + MSC^CCR2^ (1.0 × 10^6^). All infusions were performed 12 h after intraperitoneal injection of TAA, and cells suspended in 100 µl PBS were injected via the tail vein.

### Isolation, culture, and characterization of hUC-MSCs

Human umbilical cord samples were obtained from healthy donors who provided informed consent. MSCs were isolated from Wharton's jelly of the umbilical cord and cultured as described previously [26, 27]. Briefly, shortly after full-term infant delivery, the umbilical cord was collected and stored in saline. After removal of the umbilical vein and arteries, tissues were cut into 1-mm^3^ pieces and cultured by the tissue explants adherent method in 10-cm^2^ culture dishes with complete medium consisting of low-glucose Dulbecco's modified Eagle's medium (L-DMEM; Gibco, USA, C11885500BT) and 10% fetal bovine serum (FBS; BI, Israel, 04-001-1ACS). After 1 week, the remaining tissues were carefully removed, and the adherent cells were expanded for an additional 1–2 weeks. At subconfluence (80%), cells were detached with 0.25% trypsin-EDTA and passaged at a ratio of 1:3. The final MSCs used were collected at passage 4. The harvested MSCs showed strong surface expression of CD29, CD44, CD73, CD90, CD105, and CD166 (MSC markers) but lacked CD34 and CD45 (hematopoietic markers). The multilineage differentiation capacity of MSCs was confirmed by incubation in osteogenic induction medium or adipogenic induction medium (Cyagen, China, HUXUC-90021, HUXUC-90031) according to the manufacturer's instructions.

### RNA isolation, reverse transcription, and quantitative real-time PCR

Total RNA was extracted from harvested liver tissue or cells using TRIzol reagent according to the manufacturer's instructions. Reverse transcription was performed using the PrimeScript RT Reagent Kit (Takara, Japan, RR047A). qRT-PCR was performed using LightCycler 480 SYBR Green I Master Mix (Roche, Switzerland, 04887352001) on a LightCycler 480 detection system (Roche, Switzerland). Each target mRNA level was normalized to that of 18 s or GAPDH. qRT-PCR was conducted in triplicate for each sample, and three independent experiments were performed. The sequences of the primers used in this study are listed in Additional file 1: Table S2.

### Transduction with lentiviral vectors

The lentiviral expression vectors applied in this study were constructed by and purchased from GeneCopoeia (USA) and included pLV/puro-EF1α-CCR2-SV40-eGFP and pLV/puro-EF1α-SV40-eGFP (Additional file 2: Figure S1A). Lentiviruses were prepared by transient transfection of the 293FT packaging cell line using Lipofectamine 3000 (Invitrogen, USA, L3000-015) according to the manufacturer's instructions. The supernatants containing lentiviral particles were harvested at 48 h after transfection and then filtered through a 0.45-μm-pore filter membrane. For lentiviral transduction, MSCs were exposed to lentiviruses diluted in complete medium supplemented with 8 μg/mL polybrene (Sigma-Aldrich, USA, H9268) for 12 h.

### Western blot assays

For Western blotting, total protein was harvested by lysis of liver tissue or cells in lysis buffer (KeyGen Biotech, China, KGP250) with protease inhibitors, phosphatase inhibitors, and PMSF. After centrifugation at 12,000 g for 5 min at 4 °C, the supernatant was collected as the protein lysate. The protein concentration was measured using a BCA protein assay kit (KeyGen Biotech, China, KGPBCA). The protein lysate (40 µg) was separated by SDS-PAGE and transferred to a 0.2-μm-pore PVDF membrane. The membrane was blocked with 5% BSA for 1 h at room temperature and then incubated with appropriate primary antibodies overnight at 4 °C. After that, the membrane was incubated with HRP-conjugated secondary antibodies for 1 h at room temperature, and then antigen–antibody complexes were detected with WesternBright ECL (Advansta, USA, K-12045-D50). The utilized primary and secondary antibodies are listed in Additional file 1: Table S3.

### Flow cytometry

MSCs were incubated with the appropriate antibodies for 30 min in the dark at room temperature and then analyzed by flow cytometry. All flow cytometric analyses were performed with flow cytometers (BD, USA), and the data were analyzed with FlowJo software (Tree Star, USA). The utilized antibodies are listed in Additional file 1: Table S4.

### Transwell migration assays

The migratory capacity of MSCs was assessed using a Transwell chamber system with an 8.0-μm pore size (Corning, USA, 3422). Serum-starved MSC^vector^ or MSC^CCR2^ were seeded in the upper chamber (1.0 × 10^5^/well), and the lower chamber was loaded with serum-free medium with or without hCCL2 (100 ng/mL; PeproTech, USA, 300-04) or mCCL2 (200 ng/mL; PeproTech, USA, 250-10). After 8 h of coculture, the nonmigrated cells were gently removed with a cotton swab, and the filters were fixed with 4% paraformaldehyde for 15 min and stained with 0.1% crystal violet for 30 min. The number of migrated cells was determined by counting five random fields per well under a microscope at 100×.

### MSC labeling with DiR

MSCs were detached with 0.25% trypsin-EDTA, washed twice with PBS, and then incubated with 10 µg/ml 1,1-dioctadecyl-3,3,3,3-tetramethylindotricarbocyanine iodide (DiR; AAT Bioquest, USA, AAT-22070), a near-infrared fluorescence imaging probe, for 15 min at 37 °C according to the manufacturer's instructions. Then, the DiR-labeled MSCs were washed twice with PBS to ensure complete removal of any unbound dye.

### In vivo* and *ex vivo* near-infrared fluorescence imaging*

Fluorescence imaging was performed with the In Vivo Imaging System (IVIS Lumina 2, Caliper Life Sciences, USA) equipped with a standard CCD camera, and the data were analyzed with Living Image 4.5.5 software (PerkinElmer, USA).

For in vivo fluorescence imaging, mice were anesthetized with inhaled isoflurane and then placed in the imaging chamber. Fluorescence imaging was performed at 6 h, 9 h, 12 h, 24 h, 48 h, 72 h, 96 h, and 7 d after injection of DiR-labeled MSCs.

For ex vivo fluorescence imaging, mice were sacrificed at 24 h, 48 h, 96 h, or 7 d after injection of DiR-labeled MSCs. The heart, lungs, liver, spleen, and kidneys were harvested and immediately subjected to fluorescence imaging.

### Serum biochemical analysis

At the indicated time points, blood samples were harvested from each group. The serum levels of alanine aminotransferase (ALT), aspartate aminotransferase (AST), and total bilirubin (TBil) were detected using an automated biochemical analyzer (HITACHI, Japan) according to the manufacturer's instructions.

### Histopathology and immunohistochemistry

The harvested liver tissues were fixed in 4% paraformaldehyde, embedded in paraffin, and then cut into 3-μm sections. After deparaffinization and rehydration, the sections were stained with hematoxylin and eosin (H&E). For immunohistochemical (IHC) staining, after deparaffinization, rehydration, and antigen retrieval, 3% H_2_O_2_ was used to inactivate endogenous peroxidases, and goat serum was used to block nonspecific binding sites. Then, the sections were incubated with appropriate primary antibodies overnight at 4 °C. Subsequently, the Histostain-Plus BroadSpectrum Kit (Life Technologies, USA, 859043) was used for staining. Then, the sections were counterstained with hematoxylin. The number of positive cells was determined in high-power fields (200 ×) of each liver tissue section. The utilized primary antibodies are listed in Additional file 1: Table S3.

### TUNEL assays

At the indicated time points, liver tissues were harvested from each group, snap-frozen in liquid nitrogen, embedded in OCT compound, and then cut into 7-μm cryosections. TUNEL assays were performed using TMR (Red) TUNEL Cell Apoptosis Detection Kit (Servicebio, China, G1502) on cryosections under the manufacturer’s instructions. Cell nuclei were counterstained with DAPI (Beyotime, China, C1002). The sections were observed and photographed under a fluorescence microscope (Nikon, Japan). Quantification was conducted with five random fields (200 ×) for each liver tissue section using ImageJ 1.8.0 software (NIH, USA).

### Statistical analysis

All data are presented as the mean ± SD of at least three independent experiments. Data were analyzed with one-way or two-way analysis of variance (ANOVA) or unpaired two-tailed Student's t test by SPSS 20.0 software (SPSS Inc, USA). *p* < 0.05 was considered statistically significant.

## Results

### CCL2 expression is highly upregulated in the liver of ALF patients and ALF mice

**Fig. 1:**
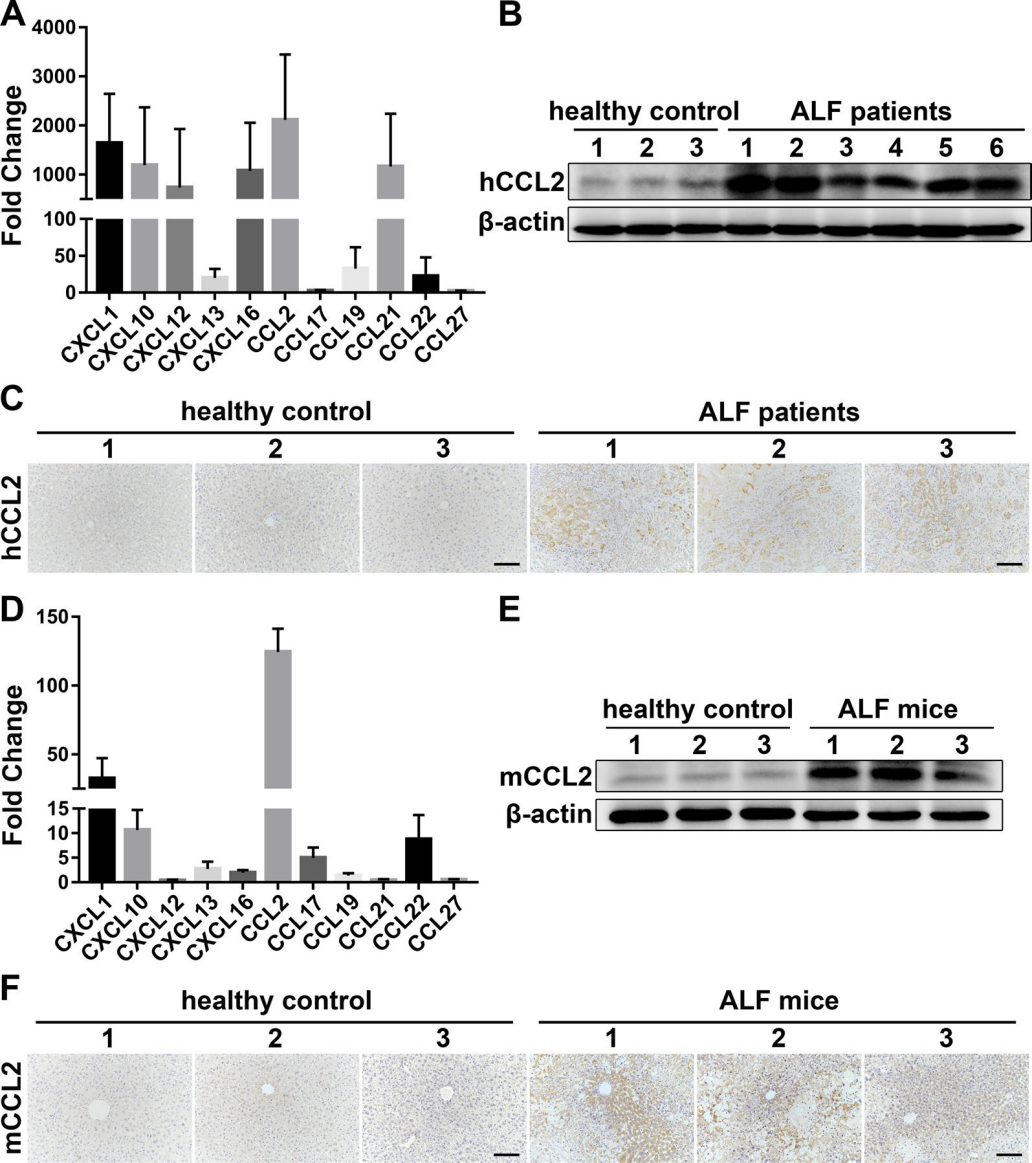
CCL2 expression is highly upregulated in the liver of ALF patients and ALF mice. **A** The expression levels of various chemokines related to liver inflammation were analyzed by qRT-PCR in the liver of healthy control and ALF patients. The fold change represents the expression of each chemokine in the liver of six ALF patients receiving liver transplantation compared to that in the liver of three healthy donors. **B** Total tissue lysates of healthy control and ALF patient livers were subjected to Western blot analysis of human CCL2 (hCCL2) protein expression. A representative blot is shown. **C** Representative anti-hCCL2 IHC staining images of liver tissue sections from healthy control and ALF patients. Scale bar: 100 µm. **D** The expression levels of various chemokines related to liver inflammation were analyzed by qRT-PCR in the liver of healthy control and ALF mice. The fold change represents the expression of each chemokine in the liver of ALF mice at 24 h after TAA injection compared to that in the liver of healthy control. *n* = 5 per group. **E** Total tissue lysates of healthy control and ALF mouse livers were subjected to Western blot analysis to assess mouse CCL2 (mCCL2) protein expression. A representative blot is shown. **F** Representative IHC images of healthy control and ALF mouse liver tissue sections stained with an anti-mCCL2 antibody. Scale bar: 100 µm. All experiments were repeated at least three times. All data are presented as the mean ± SD

To investigate the specific chemokine expression profile of ALF, we evaluated the mRNA expression levels of various chemokines associated with liver inflammation, including CXCL1, CXCL10, CXCL12, CXCL13, CXCL16, CCL2, CCL17, CCL19, CCL21, CCL22, and CCL27, in the liver of ALF patients undergoing liver transplantation and compared the levels with those in healthy donors [28–31]. The results demonstrated that the CCL2 mRNA expression level showed the most marked upregulation among the tested chemokines in the injured livers (Fig. 1A). Western blotting and immunohistochemical staining confirmed that the CCL2 protein expression level was also intensely upregulated in the liver of ALF patients (Fig. 1B, C). Similarly, the CCL2 mRNA expression level exhibited the most significant change among the aforementioned chemokines in the liver of ALF mice, and the expression level of CCL2 protein was also highly upregulated (Fig. 1D–F).

Collectively, these data indicated that CCL2 is the dominant chemokine expressed in the damaged liver.

### Chemokine receptors exhibit low-level expression in hUC-MSCs

**Fig. 2:**
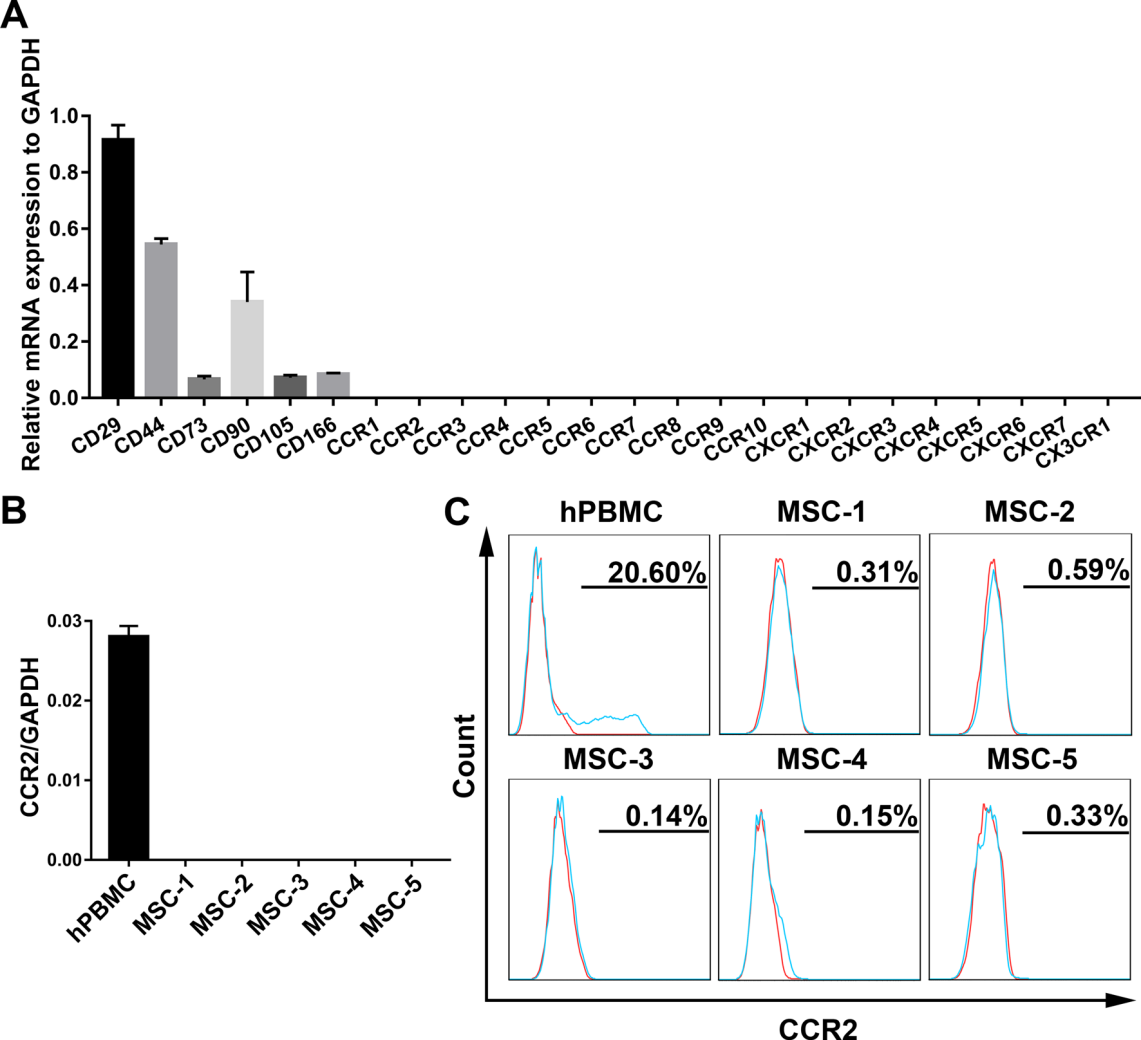
Expression profile of chemokine receptors in human umbilical cord-derived MSCs. **A** The mRNA expression levels of CC, CXC, and CX3C chemokine receptors in fourth-passage MSCs from three independent donors were analyzed by qRT-PCR. MSC surface markers (CD29, CD44, CD73, CD90, CD105, and CD166) were detected as positive controls. GAPDH served as the internal control. **B** qRT-PCR was used to detect CCR2 mRNA expression level in MSCs from an additional five independent donors, and hPBMCs served as the positive control. GAPDH served as the internal control. **C** Flow cytometry was used to detect the cell-surface CCR2 protein expression level on MSCs from five independent donors, and hPBMCs served as the positive control. All experiments were repeated at least three times. All data are presented as the mean ± SD

Chemokine receptors play a crucial role in MSC homing to injured sites by interacting with their corresponding chemokines [18–20]. Thus, we examined the expression of CC chemokine receptors (CCR1–10), CXC chemokine receptors (CXCR1–7), and a CX3C chemokine receptor (CX3CR1) through qRT-PCR analysis of MSCs from three independent donors. As shown in Fig. 2A, human umbilical cord-derived MSCs (hUC-MSCs) at the fourth passage expressed extremely low mRNA levels of chemokine receptors, including CCR2, which is the corresponding receptor of the chemokine that is highly expressed in the damaged liver, CCL2.

To confirm these data, we examined CCR2 expression on MSCs at passage 4 from an additional five independent donors. qRT-PCR analysis showed that the mRNA expression of CCR2 was low or absent in MSCs compared with human peripheral blood mononuclear cells (hPBMCs) (Fig. 2B). In addition, flow cytometric analysis further confirmed that the expression of the cell-surface CCR2 protein on MSCs was almost undetectable compared with that on hPBMCs (Fig. 2C).

Taken together, these results demonstrated that the expression of CCR2 was extremely low in culture-expanded MSCs, further suggesting that MSCs might not effectively home to the damaged liver through the CCL2/CCR2 axis.

### Genetic modification of MSCs to overexpress CCR2 without altering their intrinsic characteristics

**Fig. 3:**
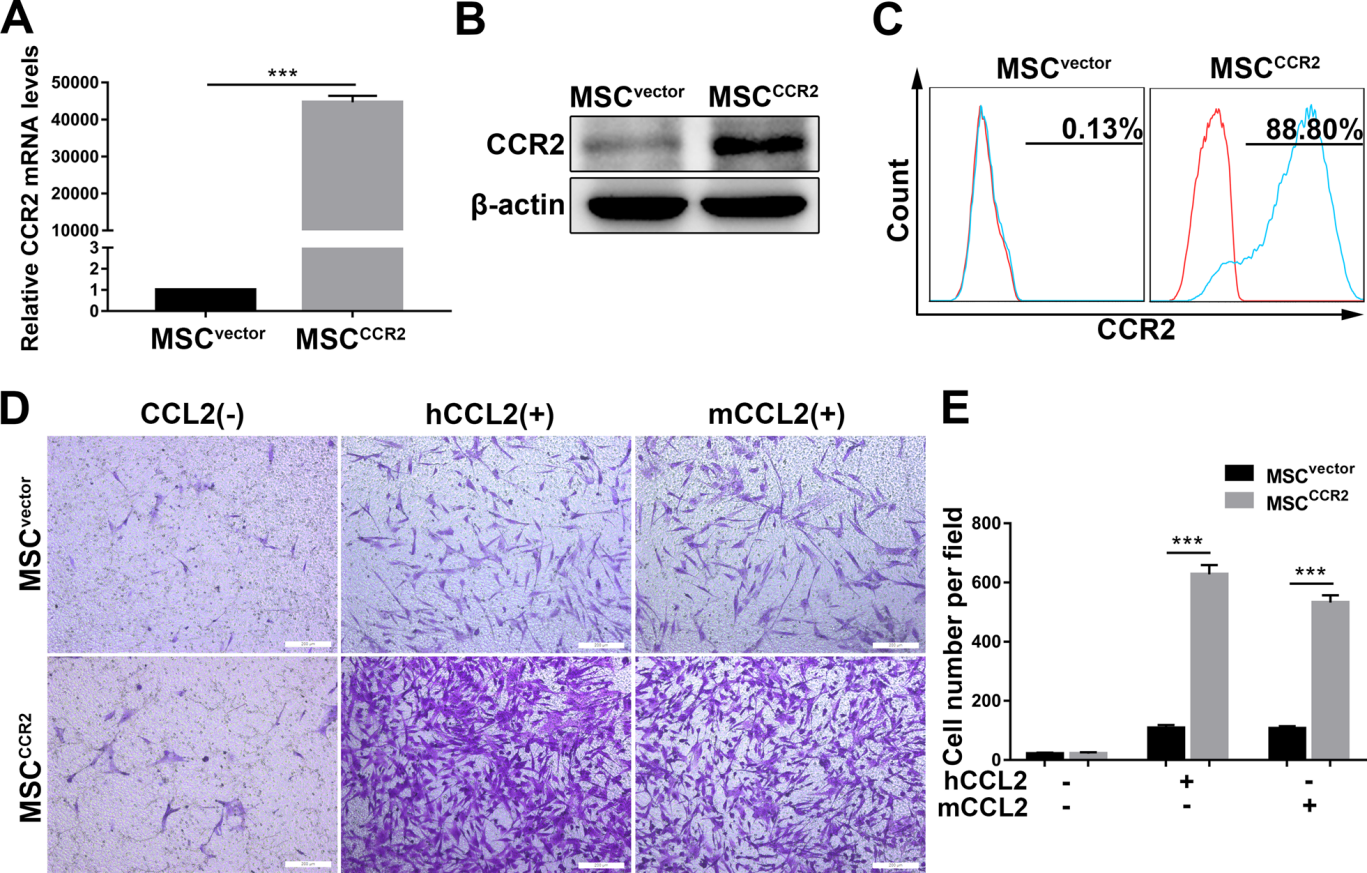
Overexpression of CCR2 in MSCs and MSC^CCR2^ exhibit enhanced migration toward CCL2 in vitro. MSCs were transduced with lentiviral vectors encoding CCR2-eGFP (referred to as MSC^CCR2^) or eGFP (referred to as MSC^vector^). **A** qRT-PCR was used to analyze the mRNA expression level of CCR2 in MSC^vector^ and MSC^CCR2^. The data are representative of three independent experiments. **B** Total cell lysates of MSC^vector^ and MSC^CCR2^ were analyzed by Western blot analysis of the CCR2 protein. The experiment was performed three times; a representative blot is shown. **C** Flow cytometry was used to analyze the level of cell-surface CCR2 protein expression on MSC^vector^ and MSC^CCR2^. The experiment was repeated three times. **D** Transwell assays were performed to detect the in vitro migration of MSC^vector^ and MSC^CCR2^ toward recombinant human CCL2 (hCCL2) or murine CCL2 (mCCL2). Transwell filters were stained with 0.1% crystal violet. Scale bar: 200 µm. **E** Quantification of migrated cells. All data are presented as the mean ± SD. *p < 0.05, **p < 0.01, ***p < 0.001, and n.s. means nonsignificant

Based on the above results, MSCs were transfected with lentiviral vectors encoding CCR2-eGFP (referred to as MSC^CCR2^) to overexpress CCR2 or eGFP (referred to as MSC^vector^) to serve as a control (Additional file 2: Figure S1A and S1B). Three days after infection, we detected remarkably upregulated expression of CCR2 at the mRNA and protein levels in MSC^CCR2^ through qRT-PCR and Western blotting, respectively (Fig. 3A, B). Flow cytometric analysis also showed that the cell-surface level of CCR2 was 88.80% on MSC^CCR2^ in comparison with 0.13% on MSC^vector^ (Fig. 3C).

Subsequently, we investigated whether genetic modification affected the biological characteristics of MSCs. We used flow cytometry to analyze the phenotype of MSCs, and the results showed that MSC surface markers (CD29, CD44, CD73, CD90, CD105, and CD166) were highly expressed and hematopoietic stem cell markers (CD34 and CD45) were absent in both MSC^vector^ and MSC^CCR2^ (Additional file 2: Figure S1C). To demonstrate the multilineage differentiation capacity of MSCs, genetically modified MSCs were cultured in osteogenic induction medium or adipogenic induction medium. As shown in Additional file 2: Figure S1D, alizarin red S staining and oil red O staining confirmed that MSC^CCR2^ exhibited no change in the osteogenic or adipogenic capacity compared with MSC^vector^. To investigate the immunoregulatory abilities of MSCs, we examined the regulatory effects of MSC^vector^ and MSC^CCR2^ on the proliferation and proinflammatory cytokine production of CD3^+^ T cells. The results showed that both MSC^vector^ and MSC^CCR2^ significantly inhibited the proliferation of CD3^+^ T cells and suppressed the percentage of TNF-α- and IFN-γ- producing CD3^+^ T cells (Additional file 3: Figure S2). Furthermore, CCR2 overexpression did not alter the proliferation and survival of MSCs, and the paracrine effects mediated by MSCs (Additional file 4: Figure S3 and Additional file 5: Figure S4).

In summary, these results indicated that hUC-MSCs were successfully modified with CCR2 and that their intrinsic characteristics were not altered.

### MSC^CCR2^ exhibit enhanced migration toward CCL2 in vitro

We next examined whether overexpression of CCR2 in MSCs could increase their migration toward the corresponding ligand CCL2. In vitro Transwell migration assays showed that MSC^CCR2^ but not MSC^vector^ forcefully responded to stimulation with recombinant human CCL2 (hCCL2) at 100 ng/mL or recombinant murine CCL2 (mCCL2) at 200 ng/ml. Additionally, in the absence of exogenous CCL2 treatment, MSC^vector^ and MSC^CCR2^ exhibited similar relatively low migration levels (Fig. 3D, E).

### MSC^CCR2^ possess an increased capacity to home to liver lesions in ALF mice in vivo

**Fig. 4:**
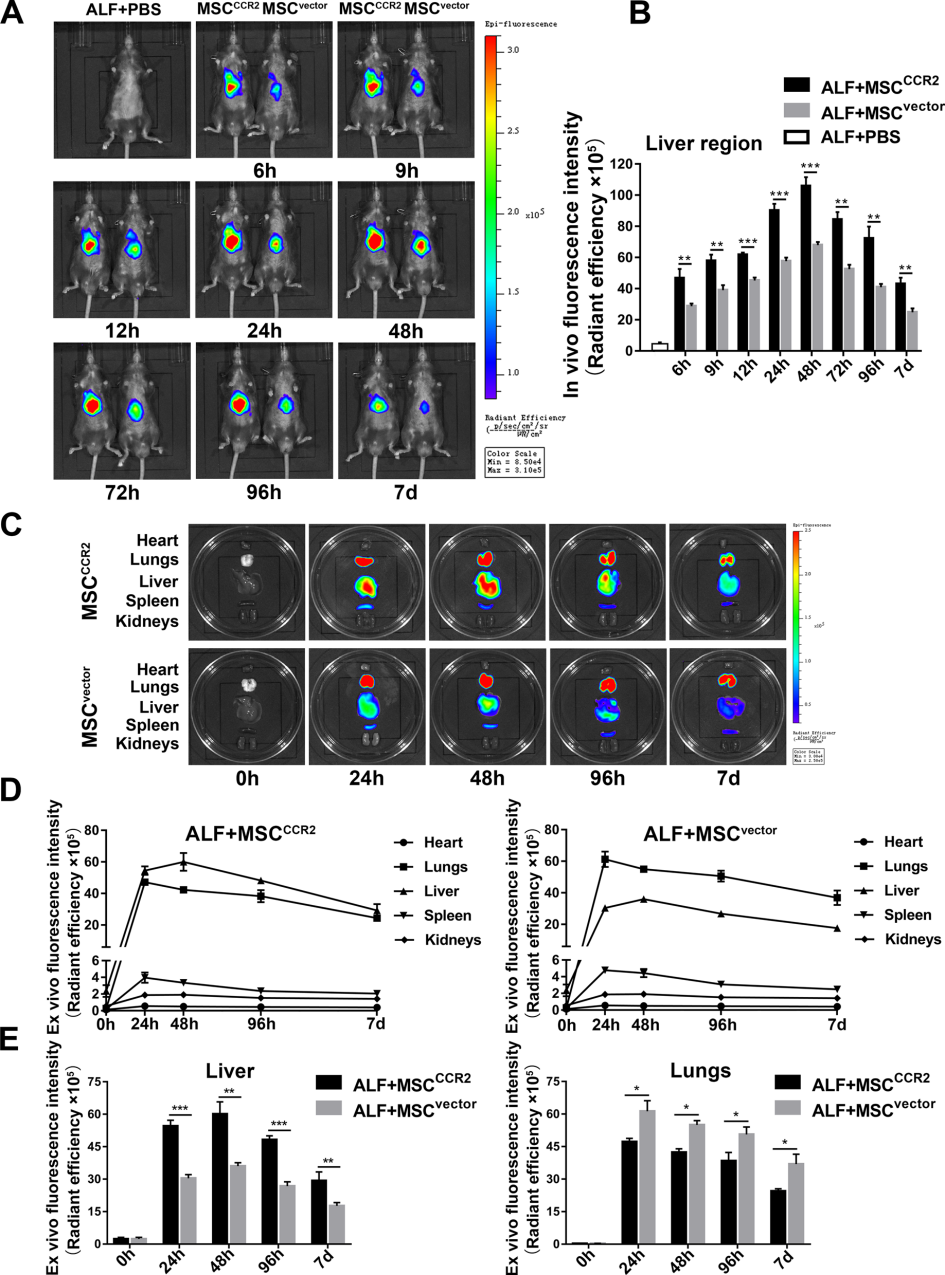
MSC^CCR2^ possess an increased capacity to home to liver lesions in ALF mice in vivo. **A** In vivo near-infrared fluorescence imaging of the MSC^vector^ group (right) and MSC^CCR2^ group (left) at 6 h, 9 h, 12 h, 24 h, 48 h, 72 h, 96 h, and 7 d after transplantation. The fluorescence signal of the PBS group served as the negative control. **B** Quantitative analysis of the fluorescence signal intensity of the liver region. *n* = 5 per group. **C** Ex vivo near-infrared fluorescence imaging of the heart, lungs, liver, spleen, and kidneys in the MSC^vector^ group and MSC^CCR2^ group at 24 h, 48 h, 96 h, and 7 d after transplantation. The fluorescence signal before transplantation (0 h) served as the negative control. **D** The line chart shows the changes in the fluorescence signal intensity of heart, lungs, liver, spleen, and kidneys over time. *n* = 5 per group. **E** Quantitative analysis of the fluorescence signal intensities of the lungs and liver. *n* = 5 per group. All data are presented as the mean ± SD. *p < 0.05, **p < 0.01, ***p < 0.001, and n.s. means nonsignificant

To determine whether MSC^CCR2^ have an enhanced ability to home to the injured liver in vivo, we infused DiR-labeled MSC^vector^ or MSC^CCR2^ into ALF mice via the tail vein and used in vivo near-infrared fluorescence imaging to examine the homing and distribution of the infused cells at 6 h, 9 h, 12 h, 24 h, 48 h, 72 h, 96 h, and 7 d after transplantation. The fluorescence signal intensity of the liver region peaked at 48 h after transplantation in both groups and then faded with time. Furthermore, the signal intensity in the MSC^CCR2^ group was remarkably higher than that in the MSC^vector^ group at each time point until 7 d after transplantation, the endpoint of observation. These data suggested that MSC^CCR2^ rapidly arrived in liver lesions at significantly greater numbers and were retained for a longer time than MSC^vector^ (Fig. 4A, B).

Then, the mice were sacrificed at 24 h, 48 h, 96 h, or 7 d after transplantation to further evaluate the five major organs in which the MSCs accumulated. The heart, lungs, liver, spleen, and kidneys were harvested, and ex vivo fluorescence imaging was performed immediately.

As shown in Fig. 4C–E, we observed that fluorescence signals were emitted from the liver, lungs, and spleen, while no signal could be detected in the heart or kidneys. The changes in the fluorescence signal intensities of the five major organs with time showed that the signal intensity of the liver in the MSC^CCR2^ group increased more sharply than that in the MSC^vector^ group, peaked at 48 h after transplantation (60.07 ± 5.67 versus 36.03 ± 1.56, *p* < 0.01), and then gradually declined until 7 d after transplantation (29.31 ± 4.00 versus 17.66 ± 1.52, *p* < 0.01). In line with the results of the in vivo fluorescence imaging study, the intensity of the fluorescence signal from the liver was remarkably higher in the MSC^CCR2^ group than in the MSC^vector^ group at each time point of observation. Liver tissue cryosections immunofluorescence staining further revealed that the accumulation of a significantly greater number of eGFP^+^ cells in the damaged liver of the MSC^CCR2^ group than in the MSC^vector^ group (Additional file 6: Figure S5).

In addition, the signal intensities of the lungs and spleen peaked at 24 h after transplantation and then decreased over time. The intensity of the fluorescence signal from the lungs was significantly lower in the MSC^CCR2^ group (47.24 ± 1.56) than in the MSC^vector^ group (61.27 ± 4.87, *p* < 0.05) at 24 h after transplantation, suggesting that the number of MSCs entrapped in the lungs was markedly decreased in the MSC^CCR2^ group. The MSC^CCR2^ group tended to have a lower signal intensity in the spleen than the MSC^vector^ group, but the difference was not statistically significant (Additional file 7: Figure S6). Moreover, the signal intensities of the heart and kidneys were almost unchanged.

Altogether, these data demonstrated that CCR2 overexpression enhances the targeted migration of MSCs to liver lesions in vivo.

### MSC^CCR2^ infusion improves survival and alleviates liver injury in ALF mice

**Fig. 5:**
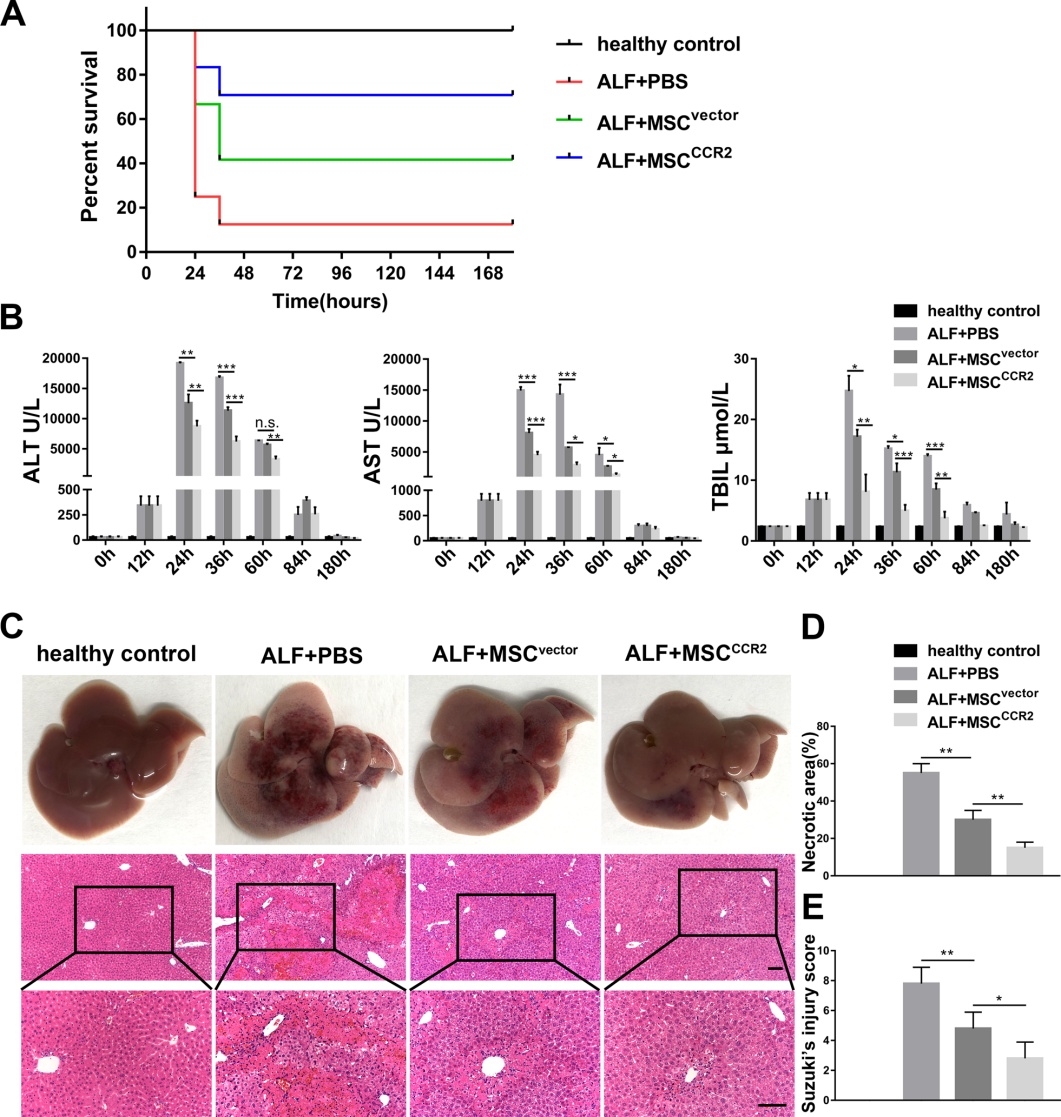
MSC^CCR2^ infusion improves survival and alleviates liver injury in ALF mice. **A** Survival analysis of each group. *n* = 24 per group. ALF + MSC^vector^ group versus ALF + PBS group, *p* < 0.05; ALF + MSC^CCR2^ group versus ALF + MSC^vector^ group, *p* < 0.05. **B** The serum levels of ALT, AST, and TBIL in each group at 0 h, 12 h, 24 h, 36 h, 60 h, 84 h, and 180 h after TAA injection. *n* = 5 per group. **C** Representative photographs and H&E staining images of liver samples from each group collected at 60 h after TAA injection. Scale bar: 100 µm. **D** Quantification of necrotic area fraction was conducted with five random fields (100 ×) for each liver tissue section. *n* = 5 per group. **E** Suzuki's injury score was used as a criterion to assess the severity of liver injury. n = 5 per group. All data are presented as the mean ± SD. *p < 0.05, **p < 0.01, ***p < 0.001, and n.s. means nonsignificant

Next, we detected whether the enhanced migration of MSC^CCR2^ to liver lesions improves the treatment effect. MSC^vector^, MSC^CCR2^, or PBS was injected into ALF mice 12 h after injection of TAA. In a survival analysis, 17/24 mice treated with MSC^CCR2^ survived until the endpoint of observation (180 h after TAA injection), fewer mice (10/24) survived in the MSC^vector^ group, and only 3/24 mice survived in the PBS group (Fig. 5A). These data showed that MSC^CCR2^ treatment significantly improved the survival rate of ALF mice.

To further evaluate the functional and histopathological changes in the liver of ALF mice, we collected serum and liver tissue samples from each group at multiple time points after TAA injection. As shown in Fig. 5B, the serum levels of alanine aminotransferase (ALT), aspartate aminotransferase (AST), and total bilirubin (TBil) in both the MSC^vector^ group and the MSC^CCR2^ group were markedly lower than those in the PBS group, and more significantly decreased levels were found in the MSC^CCR2^ group at 24 h, 36 h, and 60 h after TAA injection.

Similarly, H&E staining of liver tissue sections showed that histopathological injury was more efficiently improved in MSC^CCR2^-treated mice, which had remarkably ameliorated tissue necrosis, hepatocyte cytoplasm rarefaction, and hemorrhaging and reduced inflammatory cell infiltration, than in MSC^vector^-treated mice (necrotic area fraction: 15% ± 3% versus 30% ± 5%, *p* < 0.01; Suzuki's injury score: 2.80 ± 1.10 versus 4.80 ± 1.10, *p* < 0.05). Additionally, macroscopic injury to the liver, including swelling, congestion, and necrosis, was dramatically ameliorated in the MSC^CCR2^ group, which was consistent with the results of the histopathological study (Fig. 5C–E).

Taken together, these results indicated that MSC^CCR2^ could home to sites of liver injury with enhanced potency and more effectively alleviate liver injury.

### MSC^CCR2^ transplantation efficiently ameliorates inflammatory infiltration and hepatic apoptosis and promotes liver regeneration in the liver of ALF mice

**Fig. 6:**
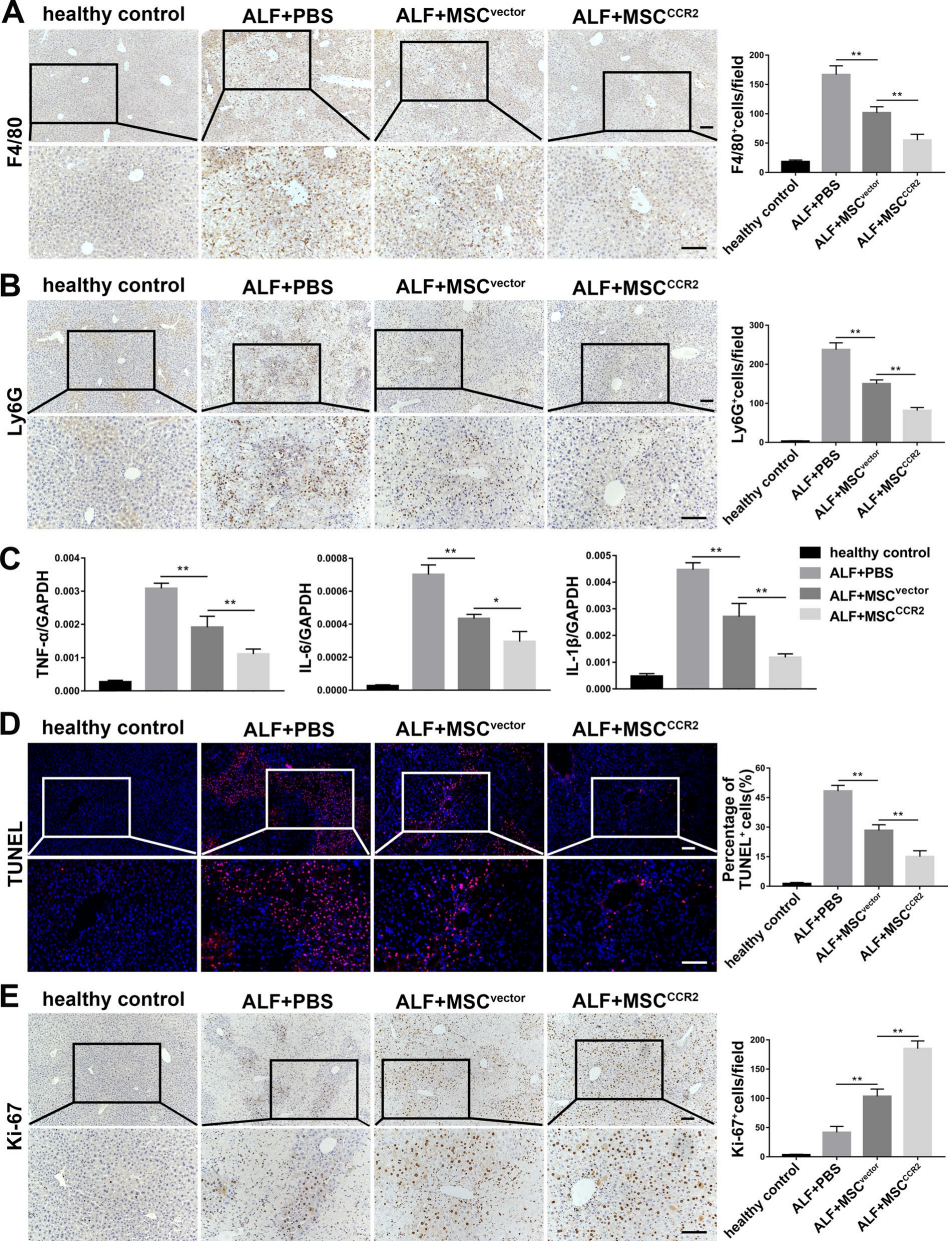
MSC^CCR2^ transplantation efficiently ameliorates inflammatory infiltration and hepatic apoptosis and promotes liver regeneration in the liver of ALF mice. **A** Hepatic macrophages level was detected using anti-F4/80 immunohistochemical staining of liver tissue sections from each group harvested at 36 h after TAA injection. Scale bar: 100 μm. Statistical analysis of the F4/80-positive cells in five random high-power fields of each section was performed. *n* = 5 per group from three independent experiments. **B** Hepatic neutrophils level was assessed by anti-Ly6G immunohistochemical staining of liver tissue sections from each group harvested at 36 h after TAA injection. Scale bar: 100 μm. Statistical analysis of the Ly6G-positive cells in five random high-power fields of each section was performed. *n* = 5 per group from three independent experiments. **C** The mRNA levels of proinflammatory cytokines TNF-α, IL-6, and IL-1β in livers from each group at 36 h after TAA injection were detected by qRT-PCR. GAPDH served as the internal control. *n* = 5 per group from three independent experiments. **D** Apoptosis level was detected using TUNEL (red) staining of liver tissue cryosections from each group harvested at 36 h after TAA injection. The nuclei were counterstained with DAPI (blue). Scale bar: 100 μm. Statistical analysis of the percentage of TUNEL-positive cells in five random high-power fields of each section was performed. *n* = 5 per group from three independent experiments. **E** Hepatocyte proliferation was assessed by anti-Ki-67 immunohistochemical staining of liver tissue sections from each group harvested at 60 h after TAA injection. Scale bar: 100 μm. Statistical analysis of the Ki-67-positive cells in five random high-power fields of each section was performed. *n* = 5 per group from three independent experiments. All data are presented as the mean ± SD. *p < 0.05, **p < 0.01, ***p < 0.001, and n.s. means nonsignificant

Inflammatory infiltration contributing to the progression of liver injury, in which hepatic macrophages and neutrophils play an important role [32–34]. Therefore, we examined the levels of macrophages and neutrophils in the liver of each group by anti-F4/80 and anti-Ly6G immunohistochemical staining, respectively. As shown in Fig. 6A and B, the PBS group displayed massive macrophages and neutrophils infiltration at 36 h after TAA injection. Meanwhile, more significantly decreased levels of hepatic macrophages and neutrophils were found in the MSC^CCR2^ group than in the MSC^vector^ group.

It might be related to the reduction of inflammation at the injured sites by MSCs through extracellular vesicles, paracrine effects, and other means. And then less inflammatory factors were produced in the alleviative damaged liver. In Additional file 8: Figure S7, some inflammatory chemokines, such as CCL2, CXCL1, and CXCL10, were highly upregulated in the PBS group by qRT-PCR analysis, and exhibited significantly decreased expression both in the MSC^vector^ group and the MSC^CCR2^ group. And more markedly reduced levels were observed in the MSC^CCR2^ group.

Furthermore, we examined the mRNA expression levels of the proinflammatory cytokines TNF-α, IL-6, and IL-1β in livers from each group. The PBS group exhibited highly increased mRNA levels of TNF-α, IL-6, and IL-1β at 36 h after TAA injection. In the meantime, the expression of the aforementioned cytokines was attenuated in both the MSC^vector^ group and MSC^CCR2^ group, with the latter exhibiting more remarkable reductions (Fig. 6C).

Finally, we evaluated the levels of apoptosis and proliferation in livers from each group by TUNEL staining and immunohistochemical staining for Ki-67, respectively. As expected, significantly decreased numbers of TUNEL^+^ cells and markedly increased numbers of Ki-67^+^ cells were observed in the MSC^CCR2^ group compared with the MSC^vector^ group (Fig. 6D, E).

Collectively, these results demonstrated that MSC^CCR2^ were preferentially able to alleviate liver injury over MSC^vector^, presumably in part due to their ability to more effectively improve the inflammatory microenvironment of the injured liver, downregulate the expression of inflammatory chemokines, attenuate hepatic inflammatory cells infiltration, suppress the production of proinflammatory factors, reduce hepatic apoptosis, and promote liver regeneration.

## Discussion

ALF is characterized by rapid progression, extensive hepatocyte necrosis, severe deterioration of liver function, and high mortality with limited therapeutic options [1]. Recently, accumulating preclinical and clinical investigations have indicated that treatment with MSCs could benefit liver injury recovery and improve survival rates [6, 7, 35].

It has been suggested that the ability of MSCs to migrate toward lesions could largely determine the treatment efficacy of these cells [9–12]. Several previous preclinical studies have indicated that intrahepatic portal vein transplantation is more beneficial than peripheral vein transplantation in ALF pigs [36, 37] and that intraperitoneal injection is superior to intravenous injection in treating colitis mice [38]. In clinical settings, some studies have directly infused MSCs into local lesions to treat patients (e.g., Crohn's disease) and achieved good treatment efficacy [39, 40]. Compared to another transplantation approach, namely, systemic administration, local implantation can facilitate sufficient numbers of MSCs localizing to injured areas. This can be explained by the pulmonary first-pass effect; that is, the majority of systemically infused MSCs become entrapped in the lungs, and only a small portion can be detected in injured tissues [15].

However, local transplantation has some adverse effects, such as additional invasive injuries and inflammation and disruption of the local microenvironment [41]. As a relatively safe method, MSC treatment by systemic administration has been common in clinical studies, although sufficient homing potency for lesions is lacking.

Hence, enhancing the targeted migration of MSCs to damaged tissues may improve the therapeutic efficiency of these cells following systemic infusion. The interactions of specific chemokines from injured tissues with the corresponding chemokine receptors expressed on the MSC surface are vital in mediating MSC migration to target lesions. Therefore, clarifying the specific chemokine expression profiles of certain diseases will provide accurate clues for improving MSC homing. Previous studies have shown that upregulating the expression of corresponding chemokine receptors in MSCs can improve the homing of these cells to damaged sites. For instance, CCR1, CCR7, or CXCR4 modification was found to enhance the migration of MSCs to the injured myocardium [42], secondary lymphoid organs [43], and infarcted myocardium [44, 45], respectively, and to promote recovery in animal disease models.

In our study, we aimed to genetically modify MSCs with certain chemokine receptors, enhance the targeted migration of MSCs toward the damaged liver, and may provide new insights into MSC therapy for ALF. Thus, the chemokine expression profile of ALF was assessed, and chemokine receptor expression in MSCs was detected. The data showed that the expression of CCL2 was greatly upregulated in the liver of ALF patients and ALF mice. CCL2, also known as monocyte chemoattractant protein-1 (MCP-1), is the main ligand for CCR2. We hypothesized that the CCL2/CCR2 axis may play a role in MSC homing, similar to its role in monocyte recruitment to sites of inflammation. Next, we found that the expression of CCR2 was extremely low in culture-expanded MSCs, which gradually lost expression of chemokine receptors during continuous in vitro passaging. Therefore, we genetically modified MSCs to overexpress CCR2 without altering their intrinsic characteristics.

As expected, in vitro Transwell migration assays showed that MSC^CCR2^ possessed a significantly enhanced ability to migrate toward CCL2 compared with MSC^vector^. In vivo and ex vivo near-infrared fluorescence imaging identified that systemically infused MSC^CCR2^ could rapidly migrate and localize to the injured liver in remarkably greater numbers and be retained for a longer time than MSC^vector^. Moreover, a significantly reduced level of lung entrapment was found in the MSC^CCR2^ group. Subsequently, we investigated the therapeutic efficacy of MSC^CCR2^ transplantation in ALF mice. Mice treated with MSC^CCR2^ displayed a significantly improved survival rate, alleviated liver damage, and accelerated liver injury repair compared with mice in the MSC^vector^ group or PBS group. Finally, we found the infiltration of hepatic macrophages and neutrophils were significantly attenuated in the MSC^CCR2^ group compared with the MSC^vector^ group. Previous studies also showed that CCR2-modified MSCs could target injured tissues, inhibiting monocytes/macrophages and neutrophils infiltration in an acute ischemic stroke rat model and a diabetic skin wound mouse model [46, 47]. It is probably related to the improvement of the inflammatory microenvironment of injured sites by MSCs through extracellular vesicles, paracrine effects, and other means. However, the potential molecular mechanism still requires further investigation. Meanwhile, the levels of proinflammatory factors including TNF-α, IL-6, and IL-1β in the liver of ALF mice were remarkably downregulated in the MSC^CCR2^ group. Moreover, the apoptosis level in the liver was significantly decreased in the MSC^CCR2^ group, while the hepatocyte proliferation level was markedly increased. As several published studies have shown that inflammatory infiltration plays an essential role in liver injury, and ameliorating inflammatory infiltration is vital for promoting recovery following ALF [48–50].

With the development of nonviral transfection methods (e.g., nanocarrier and electrotransfection), 3D culture systems, and other new technologies, cell therapy has been greatly promoted [51, 52]. We believe that targeted therapeutic strategies for different chemokine expression profiles in various diseases should be developed.

## Conclusions

In summary, we showed in this study that genetically modifying MSCs to overexpress CCR2 significantly enhanced MSC homing toward the damaged liver after systemic infusion, leading to a dramatically improved survival rate, alleviated liver injury, and accelerated liver injury recovery in ALF mice. This work may provide a strategy for targeted homing of systemically infused MSCs to liver lesions, thereby optimizing MSC treatment efficacy.

## Supplementary Information

**Additional file 1**. Supplementary Tables.

**Additional file 2**. Figure S1.

**Additional file 3**. Figure S2.

**Additional file 4**. Figure S3.

**Additional file 5**. Figure S4.

**Additional file 6**. Figure S5.

**Additional file 7**. Figure S6.

**Additional file 8**. Figure S7.

## Data Availability

All data and materials generated or used during the study are available from the corresponding authors upon reasonable request.

## Abbreviations

ALF: Acute liver failure
OLT: Orthotopic liver transplantation
hUC-MSCs: Human umbilical cord mesenchymal stem cells
hPBMCs: Human peripheral blood mononuclear cells
TAA: Thioacetamide
ALT: Alanine aminotransferase
AST: Aspartate aminotransferase
TBIL: Total bilirubin
H&E: Hematoxylin and eosin
IHC: Immunohistochemistry
qRT-PCR: Quantitative real-time polymerase chain reaction
IL: Interleukin
TNF: Tumor necrosis factor
