# Supplementary material for: CCR2-overexpressing mesenchymal stem cells targeting damaged liver enhance recovery of acute liver failure
Source: Stem Cell Res Ther. 2022 Feb 5;13:55. doi: 10.1186/s13287-022-02729-y (PMC8817567; doi:10.1186/s13287-022-02729-y)
Supplement: Supplementary file 1 — Additional file 1. Supplementary Tables. [file 13287_2022_2729_MOESM1_ESM.docx]

**Supplementary Information**

**Supplementary materials and methods**

**Immunofluorescence staining**

At the indicated time points, liver tissues were harvested from each group, snap-frozen in liquid nitrogen, embedded in OCT compound, and then cut into 7-μm cryosections. For immunofluorescence staining, 4% PFA-fixed cryosections were permeabilized with 0.25% Triton X-100 for 15 min, and then blocked with 3% BSA for 30 min. After that, the sections were incubated with the appropriate primary antibodies overnight at 4 °C, and then secondary antibodies for 1 h at room temperature. Cell nuclei were counterstained with DAPI (Beyotime, China, C1002). Images were captured with a confocal laser scanning microscope (Carl Zeiss, LSM800, Germany). Quantification was conducted with five random fields (200×) for each liver tissue section. The utilized primary and secondary antibodies are listed in Table S3.

**T lymphocyte proliferation assays**

CD3^+^ T cells were obtained from healthy donors who provided informed consent, and stained with 5 μM 5,6-carboxyfluorescein diacetate succinimidyl ester (CFSE) using a CellTrace™ CFSE Cell Proliferation Kit (Invitrogen, USA, C34570) under the manufacturer's instructions. Labeled CD3^+^ T cells were cultured with or without MSC^vector^ or MSC^CCR2^, and stimulated with 5 μg/ml phytohemagglutinin (PHA; Sigma-Aldrich, USA, L1668) for 96 h. The proliferation of CD3^+^ T cells was evaluated by flow cytometric analysis of CFSE dilutions.

**Intracellular cytokine staining**

Isolated CD3^+^ T cells were cultured with or without MSC^vector^ or MSC^CCR2^, and stimulated with 5 μg/ml PHA for 72 h. Thereafter, Leukocyte Activation Cocktail, with BD GolgiPlug™ (BD biosciences, USA, 550583) were added, and the cells were cultured for an additional 6 h. The cells were fixed, permeabilized, and stained for TNF-α and IFN-γ, and analyzed by flow cytometry.

**CCK-8 assays**

The proliferation and survival of MSC^vector^ and MSC^CCR2^ were determined using Cell Counting Kit-8 (CCK-8; Dojindo, Japan, CK04) under the manufacturer's instructions. The absorbance at 450 nm was measured using a microplate reader (BioTek, USA).

**Supplementary figure legends**

**Figure S1. CCR2 overexpression did not alter the intrinsic characteristics of MSCs.**

(A) Plasmid constructs.

(B) The green fluorescence of MSC^vector^ and MSC^CCR^^2^ was observed using fluorescence microscopy. Scale bar: 200 μm.

(C) The expression levels of CD29, CD34, CD44, CD45, CD73, CD90, CD105, and CD166 on the surface of MSC^vector^ and MSC^CCR2^ were detected by flow cytometry. The experiment was performed three times.

(D) Alizarin red S staining and oil red O staining were used to evaluate the osteogenic and adipogenic differentiation capacities, respectively, of MSC^vector^ and MSC^CCR2^. Scale bar: 200 μm; 50 μm, respectively.

**Figure S2. CCR2 overexpression did not alter the immunoregulatory abilities of MSCs.**

(A) CD3^+^ T cells were cultured with or without MSC^vector^ or MSC^CCR2^ for 96 h, and their proliferation was examined by flow cytometry. Statistical analysis of the proliferation of CD3^+^ T cells was performed.

(B) CD3^+^ T cells were cultured with or without MSC^vector^ or MSC^CCR2^ for 72 h, and their production of proinflammatory cytokines TNF-α and IFN-γ was analyzed by flow cytometry. Statistical analysis of the percentage of TNF-α- and IFN-γ- producing CD3^+^ T cells was performed.

All data are presented as the mean ± SD of three independent experiments. **p* < 0.05, ***p* < 0.01, ****p* < 0.001, and n.s. means nonsignificant.

**Figure S3. CCR2 overexpression did not alter the proliferation and survival of MSCs.**

(A) CCK-8 assays were used to detect the proliferation of MSC^vector^ and MSC^CCR2^.

(B) CCK-8 assays showed the viability of MSC^vector^ and MSC^CCR2^ after various concentrations of H_2_O_2_ treatment.

All data are presented as the mean ± SD of three independent experiments. **p* < 0.05, ***p* < 0.01, ****p* < 0.001, and n.s. means nonsignificant.

**Figure S4. CCR2 overexpression did not alter the paracrine effects mediated by MSCs.**

(A-D) The mRNA expression levels of anti-inflammatory mediators (TSG-6, IDO, COX2, and IL-10) were greatly upregulated by IFN-γ in both MSC^vector^ and MSC^CCR2^. GAPDH served as the internal control.

All data are presented as the mean ± SD of three independent experiments. **p* < 0.05, ***p* < 0.01, ****p* < 0.001, and n.s. means nonsignificant.

**Figure S5.** **MSC^CCR2^ possess an increased capacity to home to liver lesions in ALF mice in vivo.**

(A) The existence of MSC^vector^ and MSC^CCR2^, both of which expressed eGFP, was examined by immunofluorescence staining of liver tissue cryosections from each group harvested at 6 h after transplantation. The nuclei were counterstained with DAPI (blue). Scale bar: 100 µm.

(B) The number of eGFP-positive cells was determined in five random high-power fields of each section. n = 5 per group from three independent experiments.

All data are presented as the mean ± SD. **p* < 0.05, ***p* < 0.01, ****p* < 0.001, and n.s. means nonsignificant.

**Figure S6.** **Quantitative analysis of the fluorescence signal intensities of the heart, spleen, and kidneys.**

(A-C) n = 5 per group. All data are presented as the mean ± SD. **p* < 0.05, ***p* < 0.01, ****p* < 0.001, and n.s. means nonsignificant.

**Figure S7. MSC^CCR2^ infusion efficiently ameliorates inflammatory infiltration in the liver of ALF mice.**

(A) The mRNA level of CCL2 in livers from each group at 36 h after TAA injection was detected by qRT-PCR. GAPDH served as the internal control. n = 5 per group.

(B) The expression of CCL2 protein in livers from each group was evaluated by western blot analysis. The experiment was performed three times; a representative blot is shown. The gray intensity of the blots was quantified using β-actin as an internal reference.

(C-D) The mRNA levels of CXCL1 and CXCL10 in livers from each group at 36 h after TAA injection were detected by qRT-PCR. GAPDH served as the internal control. n = 5 per group.

All data are presented as the mean ± SD of three independent experiments. **p* < 0.05, ***p* < 0.01, ****p* < 0.001, and n.s. means nonsignificant.

**Supplementary tables**

**Table S1. Clinical characteristics of the ALF patients included in this study**

| N0. | Age  (years) | Gender (1/2) | Ascites | Hepatic encephalopathy | MELD score |
| --- | --- | --- | --- | --- | --- |
| 1 | 40-49 | 1 | + | + | 30 |
| 2 | 40-49 | 1 | + | + | 35 |
| 3 | 40-49 | 1 | + | + | 29 |
| 4 | 40-49 | 1 | + | + | 25 |
| 5 | 50-59 | 1 | + | + | 31 |
| 6 | 50-59 | 1 | + | + | 26 |

MELD, Model for End-stage Liver Diseases.

**Table S2. Sequence (5′ to 3′) of specific primers used for qRT-PCR** **analysis**

Human:

| Genes | Forward Sequence | Reverse Sequence |
| --- | --- | --- |
| 18s | GTAACCCGTTGAACCCCATT | CCATCCAATCGGTAGTAGCG |
| CXCL1 | CAGGGAATTCACCCCAAGAACA | GGATGCAGGATTGAGGCAAGC |
| CXCL10 | AACTGTACGCTGTACCTGCAT | ACACGTGGACAAAATTGGCTT |
| CXCL12 | ATTCTCAACACTCCAAACTGTGC | ACTTTAGCTTCGGGTCAATGC |
| CXCL13 | GCTTGAGGTGTAGATGTGTCC | CCCACGGGGCAAGATTTGAA |
| CXCL16 | GACATGCTTACTCGGGGATTG | GGACAGTGATCCTACTGGGAG |
| CCL2 | GAAAGTCTCTGCCGCCCTT | GGTGACTGGGGCATTGATTG |
| CCL17 | TCCAGGGATGCCATCGTTTT | CCTCTCAAGGCTTTGCAGGTA |
| CCL19 | CATCCCTGGGTACATCGTGAG | TCTGGATGATGCGTTCTACCC |
| CCL21 | GTTGCCTCAAGTACAGCCAAA | AGAACAGGATAGCTGGGATGG |
| CCL22 | ATTACGTCCGTTACCGTCTGC | TCCCTGAAGGTTAGCAACACC |
| CCL27 | TCAGCTCTACCGAAAGCCAC | GATGCAGATGCTGCGTTGAG |
| GAPDH | ACAACTTTGGTATCGTGGAAGG | GCCATCACGCCACAGTTTC |
| CCR1 | GACTATGACACGACCACAGAGT | CCAACCAGGCCAATGACAAATA |
| CCR2 | TGCAAAAAGCTGAAGTGCTTG | CAGCAGAGTGAGCCCACAAT |
| CCR3 | TGGCATGTGTAAGCTCCTCTC | CCTGTCGATTGTCAGCAGGATTA |
| CCR4 | CCTTGCCATCTCGGATCTGC | AGACCTAGCCCAAAAACCCAC |
| CCR5 | TTGCCAAACGCTTCTGCAAAT | AGTGGATCGGGTGTAAACTGA |
| CCR6 | TTCAGCGATGTTTTCGACTCC | GCAATCGGTACAAATAGCCTGG |
| CCR7 | CAACATCACCAGTAGCACCTGTG | TGCGGAACTTGACGCCGATGAA |
| CCR8 | CTGTCTGACCTGCTTTTTGTCT | CCACTTTGCACATTACAGTCCC |
| CCR9 | ATGTCAGGCAGTTTGCGAG | TGCAGTACCAGTAGACAAGGAT |
| CCR10 | GCAAACGCAAGGATGTCGC | CGTAGAGAACGGGATTGAGGC |
| CXCR1 | CTGACCCAGAAGCGTCACTTG | CCAGGACCTCATAGCAAACTG |
| CXCR2 | CCTGTCTTACTTTTCCGAAGGAC | TTGCTGTATTGTTGCCCATGT |
| CXCR3 | CCACCTAGCTGTAGCAGACAC | AGGGCTCCTGCGTAGAAGTT |
| CXCR4 | ACTACACCGAGGAAATGGGCT | CCCACAATGCCAGTTAAGAAGA |
| CXCR5 | CACGTTGCACCTTCTCCCAA | GGAATCCCGCCACATGGTAG |
| CXCR6 | GACTATGGGTTCAGCAGTTTCA | GGCTCTGCAACTTATGGTAGAAG |
| CXCR7 | CTATGACACGCACTGCTACATC | CTGCACGAGACTGACCACC |
| CX3CR1 | AGTGTCACCGACATTTACCTCC | AAGGCGGTAGTGAATTTGCAC |
| CD29 | GTAACCAACCGTAGCAAAGGA | TCCCCTGATCTTAATCGCAAAAC |
| CD44 | CTGCCGCTTTGCAGGTGTA | CATTGTGGGCAAGGTGCTATT |
| CD73 | CCAGTACCAGGGCACTATCTG | TGGCTCGATCAGTCCTTCCA |
| CD90 | TCACCCATCCAGTACGAGTTC | GGAGCGGTATGTGTGCTCAG |
| CD105 | GCATCCTTCGTGGAGCTACC | GAGGAGTGGTCTGGATCGG |
| CD166 | ACTTGACGTACCTCAGAATCTCA | CATCGTCGTACTGCACACTTT |

Mouse:

| Genes | Forward Sequence | Reverse Sequence |
| --- | --- | --- |
| GAPDH | AGGTCGGTGTGAACGGATTTG | GGGGTCGTTGATGGCAACA |
| CXCL1 | ACTGCACCCAAACCGAAGTC | TGGGGACACCTTTTAGCATCTT |
| CXCL10 | CCAAGTGCTGCCGTCATTTTC | TCCCTATGGCCCTCATTCTCA |
| CXCL12 | TGCATCAGTGACGGTAAACCA | CACAGTTTGGAGTGTTGAGGAT |
| CXCL13 | ATTCTGGAAGCCCATTACACA | TTTGGCACGAGGATTCACAC |
| CXCL16 | ACCCTTGTCTCTTGCGTTCTT | CAAAGTACCCTGCGGTATCTG |
| CCL2 | TAAAAACCTGGATCGGAACCAAA | GCATTAGCTTCAGATTTACGGGT |
| CCL17 | TACCATGAGGTCACTTCAGATGC | GCACTCTCGGCCTACATTGG |
| CCL19 | ACCACACTAAGGGGCTATCAG | TTCTTCAGTCTTCGGATGATGC |
| CCL21 | ACCAAGTTTAGGCTGTCCCAT | ACTTAGAGGTTCCCCGGTTC |
| CCL22 | CTCTGCCATCACGTTTAGTGAA | GACGGTTATCAAAACAACGCC |
| CCL27 | GGTACAGTCCCTTGGAGCCT | GACTGTCACCTCCAGGCTGT |
| TNF-α | GGTCTGGGCCATAGAACTGA | CAGCCTCTTCTCATTCCTGC |
| IL-6 | TGATGGATGCTACCAAACTGGA | TCTGTGACTCCAGCTTATCTCTTG |
| IL-1β | GAAATGCCACCTTTTGACAGTG | TGGATGCTCTCATCAGGACAG |

**Table S3. Primary and secondary antibodies**

| Product | Catalogue Number | Supplier |
| --- | --- | --- |
| WB:  Primary antibodies:  rabbit anti-human MCP1 | ab151538 | Abcam |
| rabbit anti-mouse MCP1 | ab25124 | Abcam |
| rabbit anti-human CCR2 | 12199S | Cell Signaling Technology |
| mouse anti-β-actin | BM0627 | Boster |
| Secondary antibodies: |  |  |
| anti-rabbit IgG HRP-linked Ab | AS014 | ABclonal |
| anti-mouse IgG HRP-linked Ab | AS003 | ABclonal |
| IF:  Primary antibody:  rabbit anti-GFP  Secondary antibody:  Alexa Fluor 488 anti-rabbit IgG  IHC: | bs-0890R  A11008 | Bioss  Invitrogen |
| Primary antibodies:  mouse anti-human MCP1 | ab9858 | Abcam |
| rabbit anti-mouse MCP1 | ab25124 | Abcam |
| rabbit anti-mouse F4/80 | 70076S | Cell Signaling Technology |
| rabbit anti-mouse Ly6G | ab238132 | Abcam |
| rabbit anti-mouse Ki67 | ab15580 | Abcam |

**Table S4. Antibodies for flow cytometry analysis**

| Product | Catalogue Number | Supplier |
| --- | --- | --- |
| APC anti-human CCR2 | 357207 | BioLegend |
| PE anti-human CD29 | 303003 | BioLegend |
| PE/Cy7 anti-human CD34 | 343515 | BioLegend |
| APC anti-human CD44 | 338805 | BioLegend |
| PE/Cy7 anti-human CD45 | 368531 | BioLegend |
| PE anti-human CD73 | 344003 | BioLegend |
| APC anti-human CD90 | 328113 | BioLegend |
| PE anti-human CD105 | 323205 | BioLegend |
| PE anti-human CD166  PE anti-human TNF-α  APC anti-human IFN-γ | 343903  554513  554702 | BioLegend  BD biosciences  BD biosciences |
