# Supplementary figures and images for: CCR2-overexpressing mesenchymal stem cells targeting damaged liver enhance recovery of acute liver failure
Source: Stem Cell Res Ther. 2022 Feb 5;13:55. doi: 10.1186/s13287-022-02729-y (PMC8817567; doi:10.1186/s13287-022-02729-y)

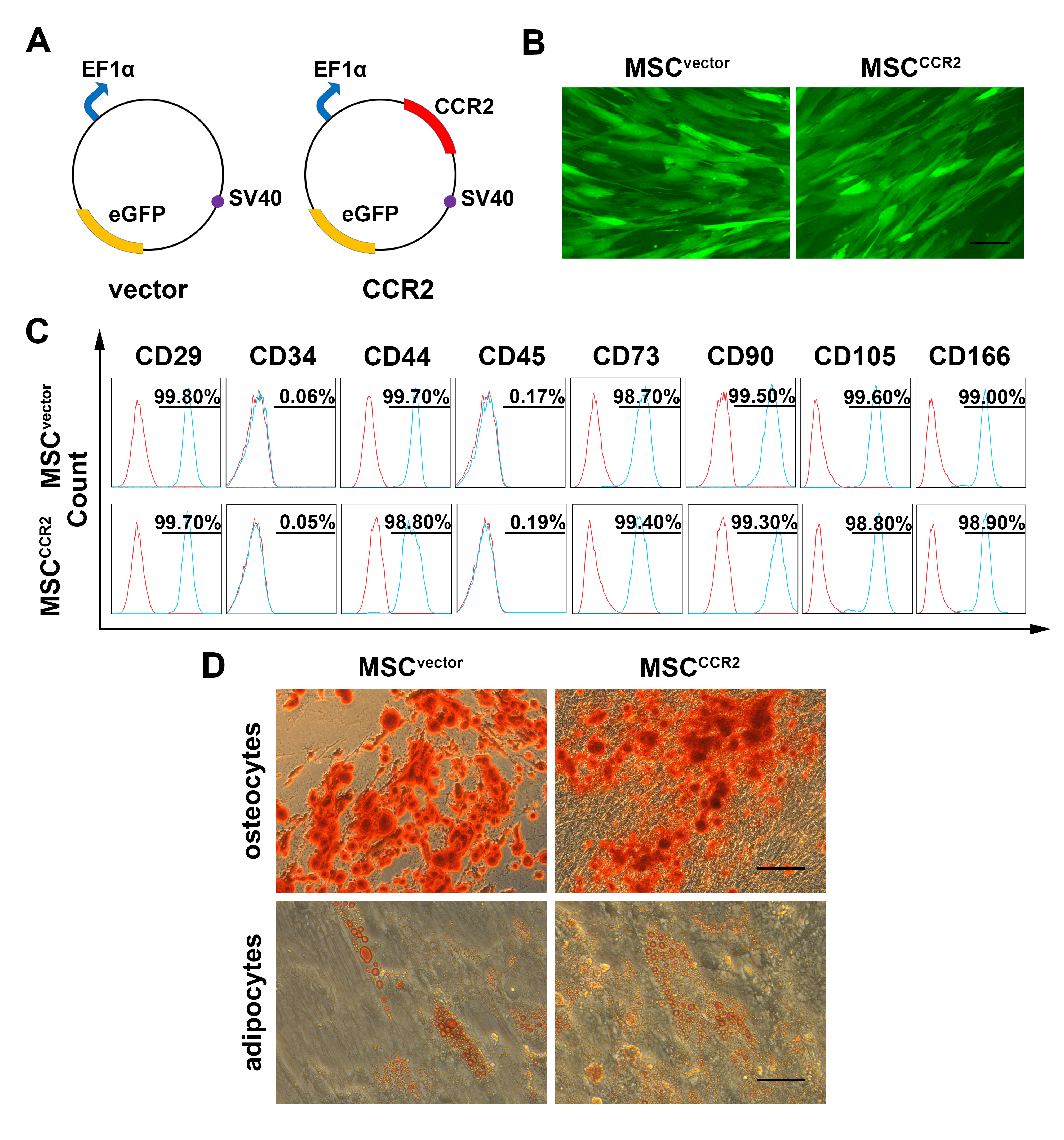

Supplement: Supplementary file 2 — Additional file 2. Figure S1. [file 13287_2022_2729_MOESM2_ESM.tif]

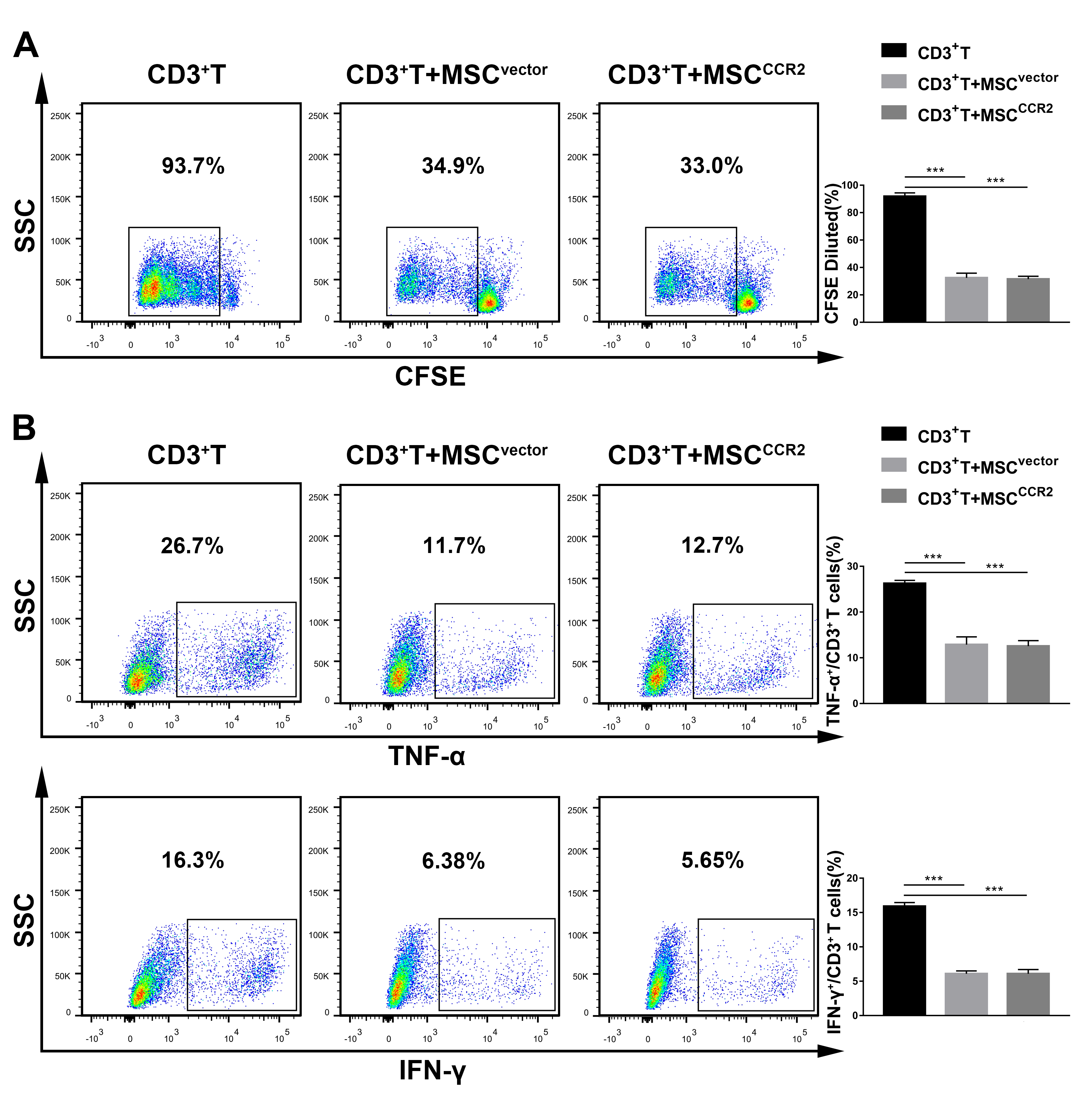

Supplement: Supplementary file 3 — Additional file 3. Figure S2. [file 13287_2022_2729_MOESM3_ESM.tif]

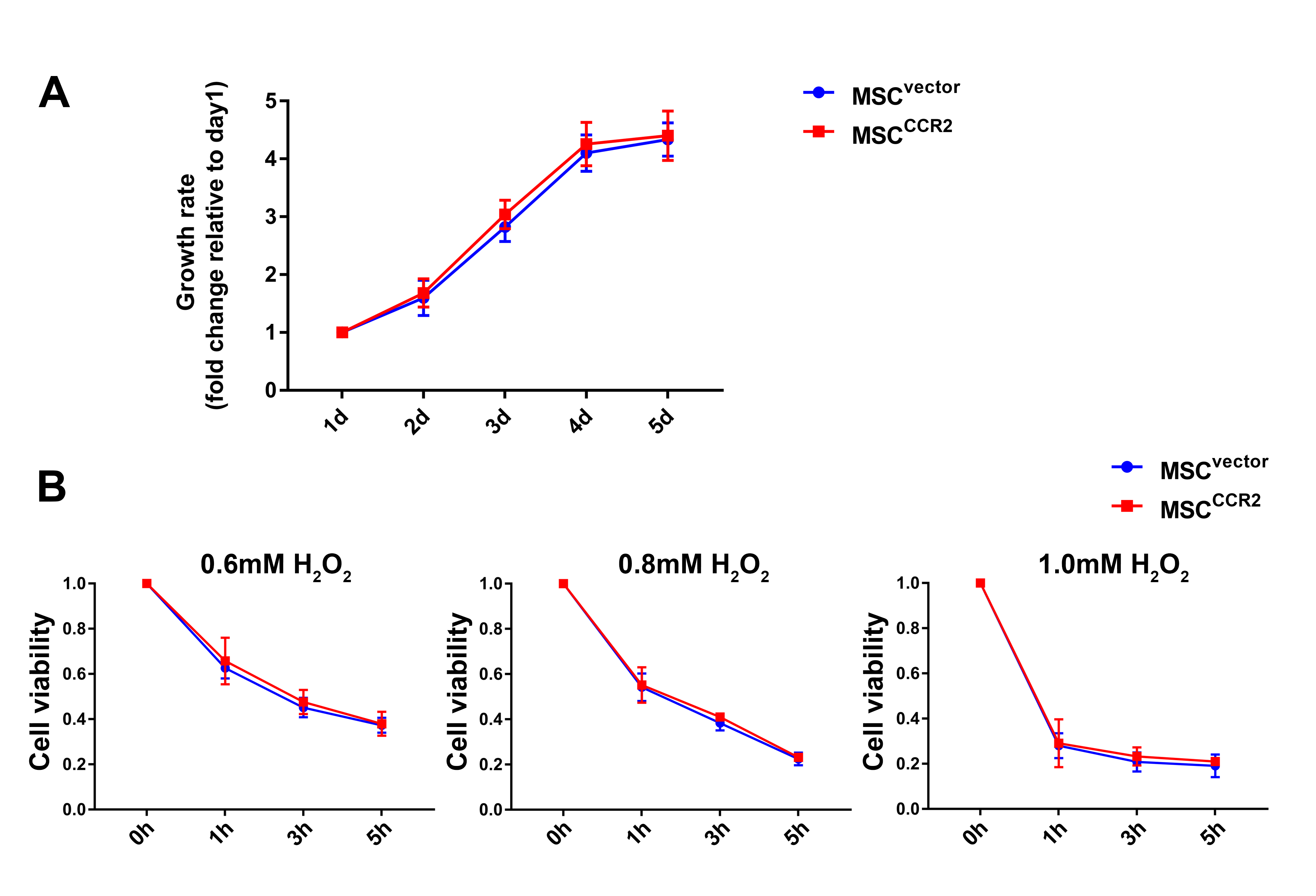

Supplement: Supplementary file 4 — Additional file 4. Figure S3. [file 13287_2022_2729_MOESM4_ESM.tif]

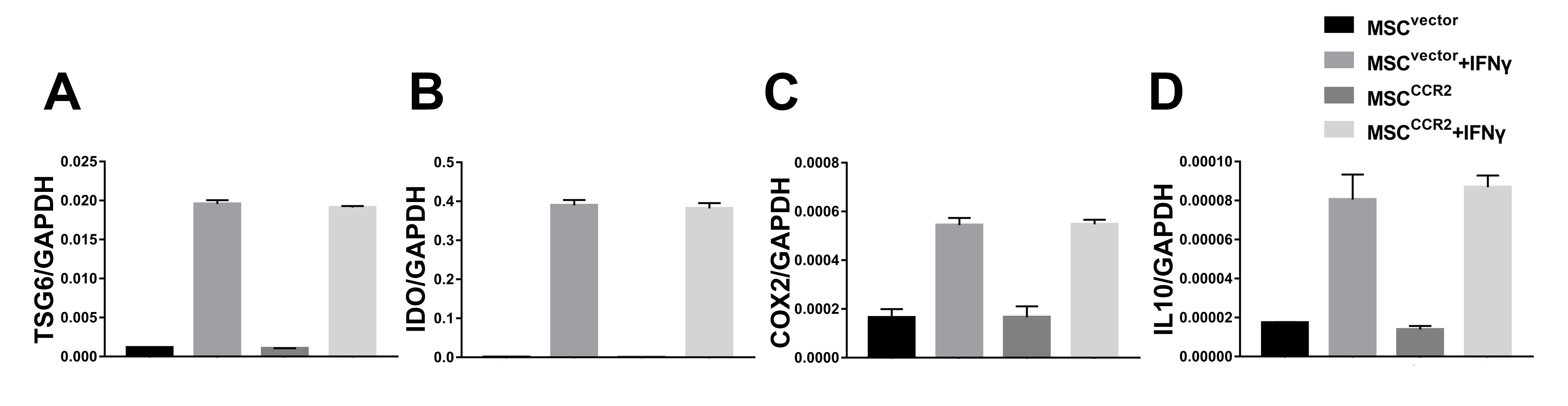

Supplement: Supplementary file 5 — Additional file 5. Figure S4. [file 13287_2022_2729_MOESM5_ESM.tif]

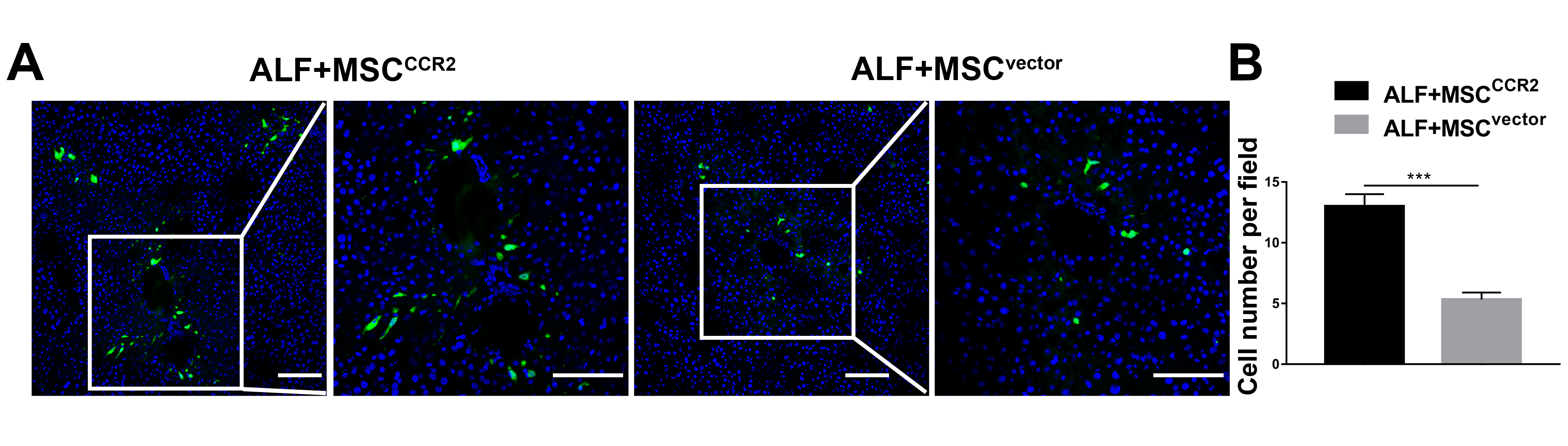

Supplement: Supplementary file 6 — Additional file 6. Figure S5. [file 13287_2022_2729_MOESM6_ESM.tif]

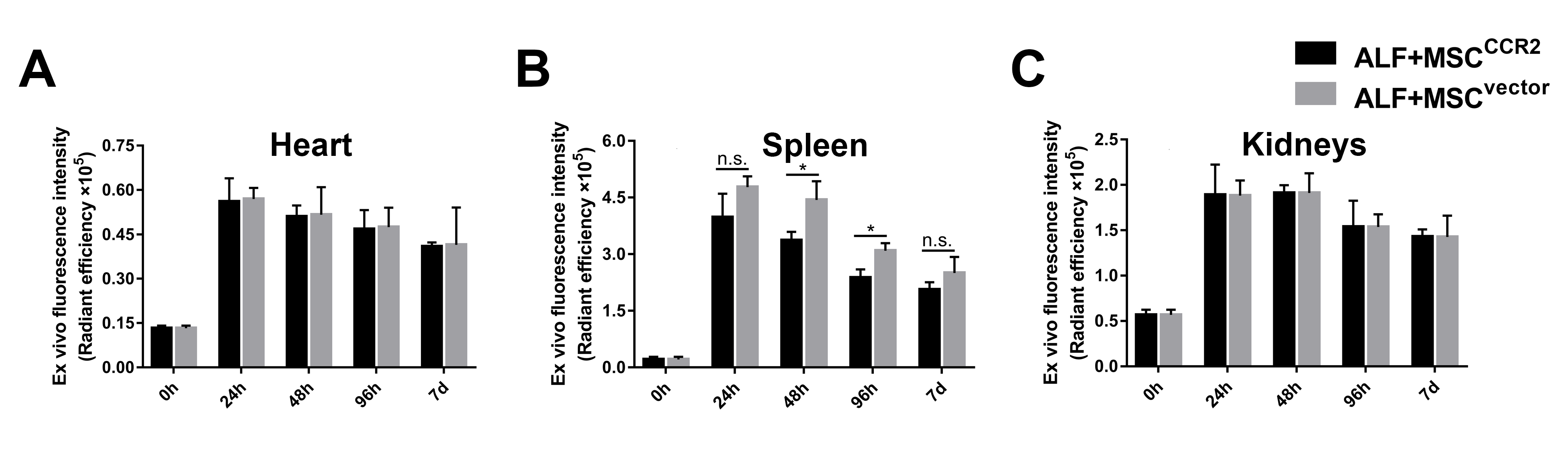

Supplement: Supplementary file 7 — Additional file 7. Figure S6. [file 13287_2022_2729_MOESM7_ESM.tif]

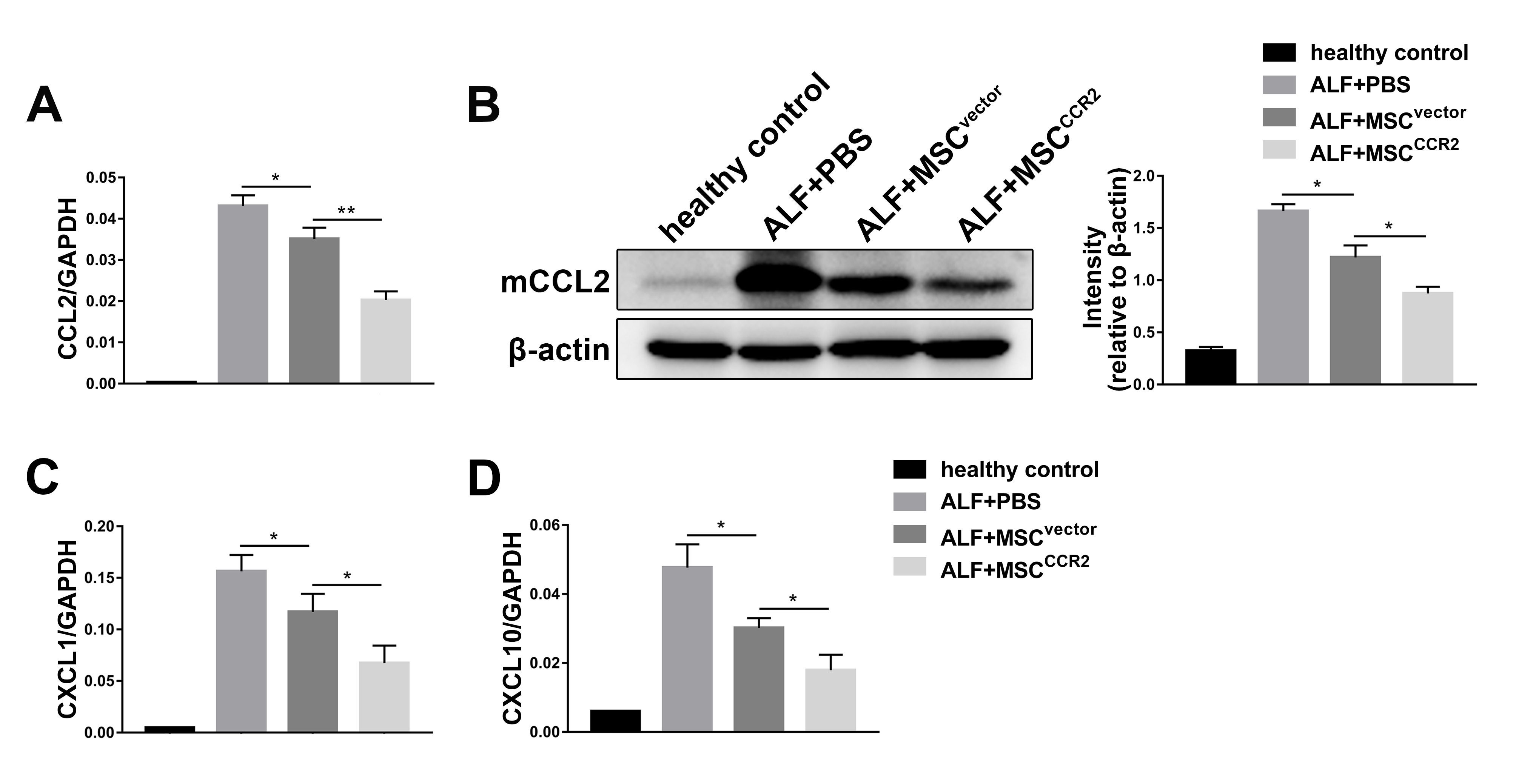

Supplement: Supplementary file 8 — Additional file 8. Figure S7. [file 13287_2022_2729_MOESM8_ESM.tif]
